# Using a Participatory Approach to Develop Research Priorities for Future Leaders in Cancer-Related Precision Public Health

**DOI:** 10.3389/fgene.2022.881527

**Published:** 2022-06-09

**Authors:** Megan C. Roberts, June Mullaney Mader, Erin Turbitt, Amelia K. Smit, Latrice Landry, Dana Lee Olstad, Lauren E. Passero, Caitlin G. Allen

**Affiliations:** ^1^ Division of Pharmaceutical Outcomes and Policy, University of North Carolina, Chapel Hill, NC, United States; ^2^ GOFORWARD LLC, Durham, NC, United States; ^3^ Graduate School of Health, University of Technology Sydney, Ultimo, NSW, Australia; ^4^ Daffodil Centre, A Joint Venture with Cancer Council NSW, The University of Sydney, Sydney, NSW, Australia; ^5^ Melanoma Institute Australia, The University of Sydney, Sydney, NSW, Australia; ^6^ Program for Cancer Care Equity, Dana Farber Cancer Institute, Boston, MA, United States; ^7^ Department of Community Health Sciences, Cumming School of Medicine, University of Calgary, Calgary, AB, Canada; ^8^ Department of Public Health Sciences, College of Medicine, Medical University of South Carolina, Charleston, SC, United States

**Keywords:** precision public health, research priorities, cancer, conference, transdisciplinary research, equity, implementation science, evaluation

## Abstract

Precision public health is an emerging discipline combining principles and frameworks of precision health with the goal of improving population health. The development of research priorities drawing on the strengths of precision and public health is critical to facilitate the growth of the discipline to improve health outcomes. We held an interactive workshop during a virtual conference bringing together early-career researchers across public health disciplines to identify research priorities in precision public health. The workshop participants discussed and voted to identify three priority areas for future research and capacity building including 1) enhancing equity and access to precision public health research and resources, 2) improving tools and metrics for evaluation and 3) applying principles of implementation science to support sustainable practices. Participants also developed future objectives for achieving each priority. Future efforts by working groups will continue the process of identifying, revising, and advancing critical research priorities to grow the impact of precision public health.

## 1 Introduction

Genomic information can personalize prevention and treatment strategies across many therapeutic areas, leading to better clinical and population health outcomes. Genomics is a cornerstone of precision medicine, which can be used along with other individual-level behaviors and environmental factors to deliver the right care to the right *patient* at the right time. Expanding these approaches to improve population health has been termed “precision public health,” the goal of which is to ensure that prevention and control strategies are delivered to the right *populations* at the right time (Khoury et al., 2016).

Numerous applications of precision public health provide opportunities to improve care. For example, newborn testing for rare diseases offers the opportunity to intervene early for treatable conditions. The use of polygenic risk scores allows precision medicine approaches for cardiovascular disease prevention. Genomic sequencing in COVID-19 surveillance enables professionals to track variants of public health concern and respond with targeted testing and vaccination in populations of greatest need (Khoury and Holt, 2021).

A key opportunity for precision public health lies in the application of genomic information to enhance cancer prevention and treatment. Despite evidence in favor of integrating genomics into population-level cancer prevention and control approaches through the use of precision public health strategies, there has been limited translation of this approach into public health and clinical practice (Roberts et al., 2019, 2017). Prior works have identified a need for research prioritization in this emerging discipline that draws from the respective strengths of precision medicine and public health to capitalize on opportunities to improve population health (Allen et al., 2019a, 2019b; Roberts et al., 2021).

Given that the field of precision public health is still emerging, there are limited opportunities for investigators to come together to form collaborations and develop much needed transdisciplinary research priorities. Instead, researchers are often siloed in their specific institution or department and may need to attend a variety of disciplinary conferences that are only tangentially related to their research agendas to learn about precision public health. To address this need, we convened an international transdisciplinary conference on October 14–15, 2021 for leaders and early-stage investigators who work in precision public health to develop and support capacity building in precision public health research, with a broad focus on oncology. During the conference, we held a workshop for participants from across different precision public health disciplines to identify transdisciplinary research priorities for precision public health in oncology.

## 2 Methods

### 2.1 Conference

*The Transdisciplinary Conference for Future Leaders in Precision Public Health* was held on October 14–15, 2021 virtually (PharmSci, 2021). Participants included international, early career researchers and practitioners as well as others interested in the topic of precision public health. The conference was advertised through social media (Twitter, San Francisco, CA), listservs, emails and all speaker and planning member networks to invite a wide audience working in diverse areas of precision public health. The conference included several key components 1) talks from a keynote speaker and six additional leaders across areas of precision public health such as environmental health, biostatistical modeling, healthy policy and health behavior, 2) networking sessions, 3) a virtual poster session held *via* Twitter and conference website (PharmSci, 2021), and 4) a workshop to identify priorities for precision public health research, which is the focus of this paper. A visual diagram of the conference proceedings is found in Supplementary Figure S1.

### 2.2 The Workshop

The conference workshop was designed to meet the following goals: 1) generate and facilitate the development of research priorities that address the challenges of and gaps in precision public health research, 2) achieve consensus about broad research priority areas, and 3) develop transdisciplinary networks. There were three workshop sessions, with a total duration of 4.5 h, distributed over the 2 days of the conference. These sessions included 1) *An “Envisioning Success” brainstorming process to identify research priority ideas*, 2) Voting by participants *on the research priority ideas to generate research priorities*, and 3) *Small group meetings to develop and draft objectives for the identified research priorities*. A professional facilitator led the sessions using the Zoom functionalities of Chat and Breakout Rooms to engage the virtual participants (Zoom Video Communications, San Jose CA). Each session began with a participant self-introduction and sharing responses to a Networking question.

#### 2.2.1 Session 1

**FIGURE 1:**
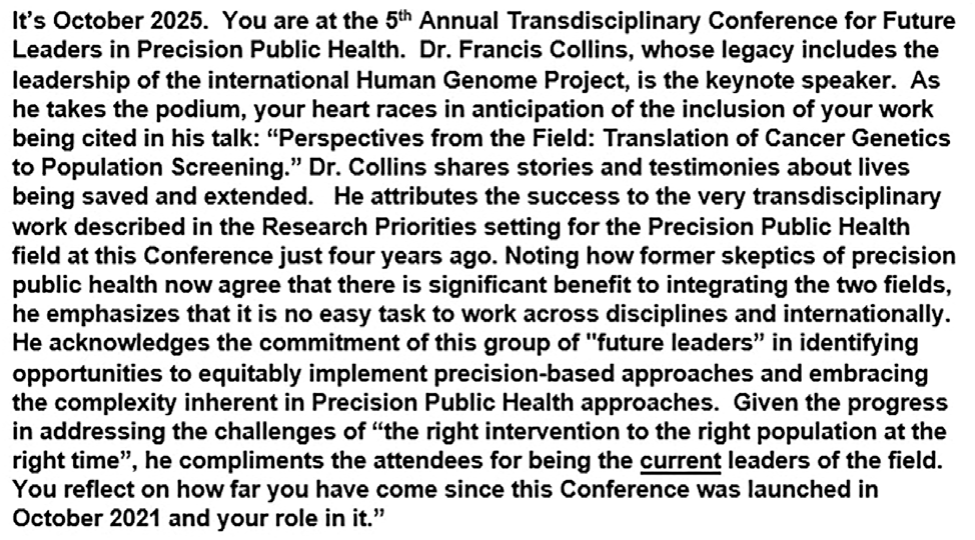
Envisioning success scenario from workshop session 1.

An appreciative-inquiry approach (Cooperrider and Whitney, 2005) was adopted to engage the participants in envisioning future success. The following “Envisioning Success” scenario was provided to stimulate the discussion (Figure 1).

Participants were then invited to individually reflect on two questions related to this success scenario and enter their responses in the Zoom chat:

1) What key research challenges did we address in defining our research priorities?
2) How did we collectively pursue these priorities?

The participants were then distributed to Zoom breakout rooms to brainstorm and identify research priorities to move the field of precision public health forward. This process involved two discussion periods in sequence, each lasting 45 min. The output from breakout discussion was the input for consideration during a larger group discussion.

The breakout session was designed to be discipline specific, to initiate the discussion among participants with a common perspective. Breakout rooms were defined for five specific public health disciplines (Health Behavior, Epidemiology, Health Policy, Biostatistics, and Environmental Health). The participants were directed to select the option that best represents their work.

The second discussion period was a full group discussion during which the output from the breakout discussions was reported to the Conference Planning Team and discussed as a larger group. The purpose of the second discussion period was to bring participants across disciplines together to develop transdisciplinary priorities.

Priorities identified in the workshop were analyzed by the Conference Planning Team immediately following session 1. Overarching concepts were developed from the priorities that had been identified by conference participants and mapped to research directions previously published by the authors (Roberts et al., 2021). These common concepts were used to categorize all research priorities and generate a list of 10 emergent research priorities based on frequency of concepts.

#### 2.2.2 Session 2

The 10 emergent research priorities were presented to all participants, who were given the opportunity to vote for their top three priorities *via* a link to an online Poll Everywhere survey (Poll Everywhere, San Francisco, CA). The survey remained open for all conference attendees and speakers to complete until four hours prior to Session 3 to accommodate participants in different time zones. We reported the percent of individuals who voted for each theme, and the top three research priorities were the basis for Session 3.

#### 2.2.3 Session 3

In this session, participants were charged with developing draft objectives to accomplish the top-three ranked research priorities.

Zoom breakout rooms were defined for each of these research priorities and the participants were asked to join the Breakout Room which best aligned with their interests. A template was provided to support the development of objectives, specifying content expectations for SMART Objectives: Specific, Measurable, Action-oriented, Realistic, Time-bound (Doran, 1981). These SMART objectives will be the foundation for ongoing research priority working groups consisting of and led by workshop participants.

## 3 Results

### 3.1 Participants

In total, 112 participants registered for the conference and 52 individuals participated during live sessions (Table 1). We advertised the availability of recorded speaker sessions for viewing on-demand at later times, so some individuals registered to receive access to recorded sessions without planning to attend live sessions. Among the 52 attendees, 15 participated in the interactive workshop sessions. All fields of public health were represented except for environmental health. Workshop participants came from Australia, South America and North America.

**TABLE 1:** Conference participant characteristics.

Characteristic	All registrants *N* (%)	Conference attendees *N* (%)	Workshop attendees *N* (%)
Geography			
Asia	1 (0.89%)	1 (1.92%)	0 (0%)
Australia	11 (9.82%)	4 (7.69%)	2 (13.33%)
Europe	1 (0.89%)	0 (0%)	0 (0%)
North America	97 (86.61%)	45 (86.54%)	12 (80.00%)
South America	2 (1.79%)	2 (3.85%)	1 (6.67%)
Public health discipline^a^			
Health Behavior	36 (32.14%)	15 (28.85%)	5 (33.33%)
Epidemiology	30 (26.79%)	17 (32.69%)	5 (33.33%)
Health Policy	43 (38.39%)	15 (28.85%)	5 (33.33%)
Biostatistics	14 (12.50%)	7 (13.46%)	2 (13.33%)
Environmental Health	9 (8.04%)	7 (13.46%)	0 (0%)
Total	112 (100%)	52 (46.43%)	15 (13.39%)

a Public Health Discipline was self-reported by participants and more than one discipline could be selected.

### 3.2 Sessions 1 and 2: Research Priority Areas

The research priority ideas generated in Session 1 as most important to move the field of precision public health forward and build capacity for precision public health included equity and access, evaluation, research capacity, infrastructure, implementation research, workforce preparation, stakeholder engagement, public education, collaboration and ethical considerations (Table 2). Equity and access, evaluation and implementation science were ranked as the top three priorities for precision public health.

**TABLE 2:** Precision public health research priorities: themes from breakout session one.

Research priority	Description	% votes *n* = 28
Equity and access	Increase the diversity of participants included in precision public health research so that everyone has access to it	19
Evaluation	Standardize evaluation of precision public health interventions and research (e.g. clinical utility, cost-effectiveness, and patient-reported outcomes)	21
Research capacity	Advance training, mentorship and opportunities for researchers at all levels (particularly early career) in precision public health	8
Infrastructure	Identify data sources, leverage existing databases and improvements in how to store, access and link data from multiple sources	9
Implementation research	Support delivery and long-term sustainability of precision public health research initiatives and interventions	15
Workforce preparation	Prepare health professionals to deliver precision public health interventions, including appropriate training and education	8
Stakeholder engagement	Involve stakeholders (e.g., communities, payers etc.) at all stages of precision public health research	10
Public education	Increase public understanding of precision public health, genetic and genomic risk communication	5
Collaboration	Advance transdisciplinary and cross-industry partnerships in tackling precision public health challenges	4
Ethical considerations	Advance understanding about key ethical considerations in precision public health	1

### 3.3 Session 3: Objectives for Research Priority Areas

Due to time constraints participants were not able to fully develop SMART Objectives. Here we report draft objectives that will continue to be developed into SMART objectives.

#### 3.3.1 Equity and Access

The overarching aim of this priority area, as discussed in Sessions 1 and 2, was *to increase the diversity, equity, and inclusion of participants in precision public health research so that everyone has access to it*. During Session 3, the subgroup discussed a need to think critically about diversity, including how it is defined and suggested working from the definition of underrepresented populations used in The *All of Us* Research Program (Mapes et al., 2020). The group proposed that additional work may be needed to identify gaps in health equity related to precision public health in order to ensure that true equity, not simply diverse recruitment, is achieved. Diversity was discussed in terms of socioeconomics, race/ethnicity, geography, as well as other dimensions of diversity yet to be identified through health equity research in precision public health. In addition, the group discussed a need to consider equity and access across the translational research spectrum, including who is included in precision public health and precision medicine research. Specific objectives primarily included objectives that were foundational to better understanding and laying the groundwork for equity research in precision public health (Table 3). Further, it was discussed that in places where known disparities in equity and access to precision public health exist (e.g., access to genetic testing for hereditary cancer conditions), work to intervene on them should advance.

**TABLE 3:** Objectives by priority.

Top three research priority areas	Preliminary objectives
Equity and Access	
	Conduct a scoping review to understand barriers for the inclusion of under-represented populations in precision public health/precision medicine research across the translational spectrum (using NIH All of US definition of under-represented populations)
	Develop a framework for evaluating whether health equity has been adequately integrated into precision public health research and interventions
	Develop a justice-based model for identifying potential harms and unintended consequences in precision public health
	Identify/implement mechanisms for promoting a diverse workforce in precision public health practice and research
Evaluation	
	Consolidate and develop tools to evaluate the effectiveness of precision public health approaches, including: an objective list of quality measures/criteria, collaborative efforts with grant review criteria, predictive measures for evaluated expected value and impact on health outcomes, identification of which predictive strategies/approaches to use, modified existing frameworks in cost-effectiveness and public health evaluation programs, evaluation tools that incorporate qualitative/mixed methods, and quantitative approaches; ways to track precision public health programs to identify where/when evaluation is needed
	Develop competencies to guide training initiatives in precision public health
	Evaluate new precision public health approaches and applications in comparison to more traditional approaches used within the field of public health (e.g., cost-effectiveness)
	Link with implementation scientists to undertake dissemination of best practices related to precision public health
	Develop metrics to evaluate the success of evaluations in guiding research and practice directions
Implementation Science	
	Promote stakeholder (e.g., community, patient, clinician, policymakers, payers) engagement and use of measures of feasibility and acceptability (ideally common measures) during pre-implementation phases of precision public health programs
	Apply implementation science to address technical needs (e.g., electronic health records) for precision public health research and practice
	Design for dissemination, develop research programs that have sustainability and spread plans (e.g., model after National Center for Advancing Translational Sciences RFA or after how Patient Centered Outcomes Research Institute mandates specific things on community engagement)
	Create a repository for findings on successful implementation strategies, sharing knowledge across settings, not limited to the high burden of creating peer reviewed literature, more rapid sharing, accessible to clinicians not just researchers
	Conduct research that evaluates implementation of precision public health iteratively and includes implementation needs in cost-effectiveness/economic modeling

#### 3.3.2 Evaluation

This group discussed how to evaluate whether “the right intervention is given to the right population at the right time” (Khoury et al., 2016). The general aim of this priority area was *to develop evaluation metrics and tools that are specific enough to be meaningful across diverse settings, and also allow for adaptation to specific settings.* Specific objectives proposed for this research priority area focused on consolidating and adapting frameworks and tools to evaluate effectiveness, develop metrics for precision public health outcomes and training initiatives that can apply to qualitative and quantitative approaches, as well as develop, evaluate, and compare new precision public health approaches to traditional methods (Table 3). Finally, the group also emphasized the need for developing standards by which to identify areas in precision public health where evaluation is most needed.

#### 3.3.3 Implementation Research

A number of specific objectives were developed to *support the delivery and long-term sustainability of precision public health using the tools of implementation science.* The group discussed sub-objectives across different phases of precision public health translation including: pre-implementation (stakeholder engagement, assessment of feasibility and acceptability), implementation (e.g., attending to technical needs) and sustainability (scale and spread, sharing knowledge, iterative evaluation) (Table 3).

## 4 Discussion

Our workshop participants developed three research priority areas for the field of precision public health: Equity and access, evaluation and implementation research. These priority areas are the foundation for ongoing working groups following the conference. While the third session aimed to create SMART objectives for each research priority, the participants ended up developing broader objectives given the available time. As a first charge, these groups will work to refine objectives to be SMART objectives and develop a set of group goals to begin advancing research in these priority areas.

### 4.1 Health Equity

Health equity has been a long-standing concern related to the field of precision public health, in particular as it relates to potential unintended consequences on existing health disparities if innovations are not accessible in an equitable manner (Korngiebel et al., 2017). At the same time, this concern for health equity has been viewed as a core reason for why precision public health research is imperative in an era of precision medicine and precision prevention. Precision public health can study issues of equity in precision public health research and practice and tailor strategies and initiatives to improve equitable access to high quality care (Roberts et al., 2021). Further, health equity must be considered not just in the implementation of precision public health, but also across the translational research pathway (Landry et al., 2018).

To date, much of the existing health equity work that has been done in precision public health has been related to issues of racial and ethnic diversity. For instance, extensive research has demonstrated racial and ethnic disparities in access to genetic testing for hereditary breast and ovarian cancer (Williams et al., 2019) as well as Lynch syndrome (Dharwadkar et al., 2022). Interventions to ameliorate these disparities must be developed with intentions to sustain and spread them across settings. Other dimensions of diversity have been less explored, for example geographic, gender, and ability diversity, and for this reason, participants in the working group believed additional work to fully understand who is at risk of falling behind in precision public health is needed.

With this knowledge in hand, justice-based frameworks and models for providing health equity in precision public health are needed. An existing framework for precision public health by Olstad and McIntyre defines precision public health as the study of how different dimensions of social position interact to shape health risk for precisely defined population groups, while also integrating relevant biological and behavioural considerations (Olstad and McIntyre, 2019). Such frameworks can serve a foundation for work by researchers and practitioners in the field of precision public health to promote health equity. Finally, a need to build capacity for precision public health research through training the next generation of precision public health researchers has been established in the literature (Allen et al., 2019a, 2019b; Pearson et al., 2020). This group called for specific attention to promoting diversity among precision public health researchers, aligning with calls for promoting diversity in genomics research more broadly (Robbins et al., 2021).

### 4.2 Evaluation

As the field of precision public health continues to develop, there is an urgent need to assess methods and common measures (both qualitative and quantitative) for precision public health research, including predictive analytics. Indeed, others in the field have noted the challenges of heterogeneous data measures and sources on their effective use for predictive analytics, as the field of precision public health advances (Pearson et al., 2020).

Developing common outcomes metrics to evaluate the use and effectiveness of precision public health is an area of recognized need (Doyle et al., 2018). In 2018, a team of multidisciplinary researchers developed common metrics for assessing the implementation of state public health programs aimed to improve Hereditary Breast and Ovarian Cancer and Lynch syndromes. The team, consisting of diverse stakeholders in implementation science, patient advocacy, medical genetics, health literacy, disparities and public health practitioners, developed 38 outcomes. As noted by the authors, additional efforts to test the validity of these outcomes and develop outcomes for other types of precision public health efforts are still needed, including standard metrics for evaluating the costs of precision public health interventions.

### 4.3 Implementation Science

Prior work has demonstrated a gap in literature and research that bridges the fields of genomic medicine and implementation science (Roberts et al., 2019, 2017). Similarly, the implementation sub-group identified a need to merge more broadly precision public health with implementation science. Other work in the field of precision public health has recognized this need as well from translating genomics into clinical settings (Veterans Health Administration, 2021; Chambers et al., 2016; Genomics, 2020) to advancing state precision public health initiatives (Doyle et al., 2018).

### 4.4 Future Directions and Limitations

Cutting across these three areas was a recognized need for increased capacity building for precision public health researchers in health equity, evaluation methods, and implementation science. Additionally, areas of overlap identified between these three priorities offer opportunities for growth of the field. For example, themes of advancing research in implementation and health equity arose and have been supported in the literature (Yousefi Nooraie et al., 2020), as well as overlap between advancing evaluation of precision public health and implementation science. A recent JACC State of the Art Review reported a need to crosscut predictive analytics methods with implementation science (Pearson et al., 2020). Thus, future work across our priority areas will be essential to drive growth in precision public health research and implementation in practice. Finally, while health equity, evaluation and implementation science were prioritized, additional priority areas were identified during Session 1. Future work should further explore research priorities within these areas as well.

These research priorities lists were developed with input from 15 engaged precision public health researchers and practitioners: Given the small number of researchers who participated in this process, we are likely missing some key perspectives. Future efforts will continue to discern valuable directions for research in precision public health and engage diverse precision public health research disciplines in these efforts through working groups. We also plan to develop the Transdisciplinary Conference for Future Leaders in Precision Public Health into an annual event bringing together researchers, clinicians, and policymakers to refine continually research directions for the field. At future conferences, we will intentionally seek increased participation from an international audience. We will engage our professional networks at the CDC Office of Genomics and Precision Public Health (CDC, 2021) and the PharmAlliance network (PharmAlliance, 2022) to conduct widespread promotion to international researchers. Additionally, we plan to offer opportunities for funding support for travel to encourage diverse participation by an international audience with specific travel awards reserved for individuals from low- and middle-income countries.

## 5 Conclusion

Health equity and access, evaluation and implementation science related objectives in precision public health have been developed and continue to be refined and cross-examined by current working groups. Next steps to address objectives raised by these groups will bring us closer to advancing the field of precision public health.

## Data Availability

The deidentified raw data supporting the conclusion of this article will be made available by the authors upon request, without undue reservation.
