# Supplementary material for: Using a Participatory Approach to Develop Research Priorities for Future Leaders in Cancer-Related Precision Public Health
Source: Front Genet. 2022 Jun 9;13:881527. doi: 10.3389/fgene.2022.881527 (PMC9218810; doi:10.3389/fgene.2022.881527)
Supplement: Supplementary file 1 [file DataSheet1.docx]

- Audience of international, early-career researchers and practitioners
- Expert speakers in precision public health across disciplines
- Twitter poster session for emerging research

**Conference**

- Increase diversity, equity, and inclusion in research
- Develop evaluation metrics and tools
- Support delivery and sustainability using implementation science

**Research Priorities**

**Workshop**

- Research priority brainstorming with participants
- Voting to identify top 3 priorities from 10 emergent themes
- Planning research objectives for top 3 priorities

Supplemental Figure 1: Visual Diagram of Conference Proceedings
